# Clostridioides Difficile Enteritis: Case Report and Literature Review

**DOI:** 10.3390/antibiotics11020206

**Published:** 2022-02-06

**Authors:** Artsiom Klimko, Cristian George Tieranu, Ana-Maria Curte, Carmen Monica Preda, Ioana Tieranu, Andrei Ovidiu Olteanu, Elena Mirela Ionescu

**Affiliations:** 1 Division of Physiology and Neuroscience, “Carol Davila” University of Medicine and Pharmacy, 050747 Bucharest, Romania; artsiom.klimko@stud.umfcd.ro; 2 Department of Gastroenterology, “Carol Davila” University of Medicine and Pharmacy, 020021 Bucharest, Romania; carmenmonica.preda@gmail.com (C.M.P.); dr.olteanuandrei@gmail.com (A.O.O.); mirela.ionescu@umfcd.ro (E.M.I.); 3 Department of Gastroenterology, “Elias” Emergency University Hospital, 011461 Bucharest, Romania; 4 Department of Pathology, “Elias” Emergency University Hospital, 011461 Bucharest, Romania; anma.pop@gmail.com; 5 Department of Gastroenterology, Fundeni Clinical Institute, 022328 Bucharest, Romania; 6 Department of Pediatrics, “Carol Davila” University of Medicine and Pharmacy, 020021 Bucharest, Romania; Ioana.tieranu1@drd.umfcd.ro

**Keywords:** Clostridioides difficile, enteritis, ileostomy, dehydration

## Abstract

Background: *Clostridioides Difficile* is a well-known pathogen causing diarrhea of various degrees of severity through associated infectious colitis. However, there have been reports of infectious enteritis mainly in patients with ileostomy, causing dehydration through high-output volume; Case presentation: We report the case of a 46-year-old male patient, malnourished, who presented with high-output ileostomy following a recent hospitalization where he had suffered an ileo-colic resection with ileal and transverse colon double ostomy, for stricturing Crohn’s disease. *Clostridioides Difficile* toxin A was identified in the ileal output confirming the diagnosis of acute enteritis. Treatment with oral Vancomycin was initiated with rapid reduction of the ileostomy output volume; Conclusion: We report a case of *Clostridioides Difficile* enteral infection as a cause for high-output ileostomy, successfully treated with oral Vancomycin. We also review the existing literature data regarding this specific localized infection.

## 1. Introduction

*Clostridioides Difficile* (CD) is a challenging global healthcare issue—CD is the leading cause of healthcare-associated infection, with a variable clinical course that ranges from mild disease to severe colitis and toxic megacolon with a 5.9% mortality rate [1]. Conventionally, CD is limited to the large bowel which has been attributed to molecular and physiologic differences between the small and large bowel [2]. However, there is increasing evidence indicating CD may also affect the small bowel, termed CD enteritis (CDE), which is associated with a protracted clinical course and mortality rates approaching 30% [3]. We present a case of CDE and conduct a literature review and pooled analysis of all documented CDE cases to provide contemporary information pertaining to patient characteristics, management consideration, and mortality rates.

## 2. Case Presentation

A 46-year-old male patient was admitted to the Gastroenterology Department of the “Elias” Emergency University Hospital in Bucharest for high-output ileostomy (approximately 1500 mL/24 h), oliguria, and diffuse colicky abdominal pain. His symptoms gradually worsened over the preceding two weeks and were accompanied by a 6 kg weight loss. He had long-standing history of neglected stricturing ileal Crohn’s disease and he had undergone laparotomy for intestinal obstruction secondary to ileal strictures several weeks prior to current hospital admission. The patient was immunocompetent, with negative molecular tests for human immunodeficiency virus. Additionally, he had HLA-B27-associated ankylosing spondylitis treated sporadically with non-steroidal anti-inflammatory drugs. His family history was negative for inflammatory bowel disease (IBD) and colorectal cancer. He denied the use of illicit substances, alcohol consumption or smoking prior to the hospital admission. Upon hospitalization, he was underweight, with a body mass index of 17 kg/m^2^.

Clinical examination upon admission revealed normal hemodynamic and respiratory parameters, normal temperature, with diffuse pain upon palpation without acute peritoneal signs.

Laboratory data showed mild leukocytosis (14.000/mmc) with neutrophilia, elevated C-reactive protein at 15-fold increase above the upper limit of normal (75 mg/dL, normal value < 5 mg/dL), hyperkalemia (6.3 mmol/L), hyponatremia (132 mmol/L), elevated serum urea (97 mg/dL) and creatinine levels (1.7 mg/dL). Ileal output obtained from the ostomy bag was used for further bacterial and parasitic testing. Ova and parasite analysis was performed via microscopy, as this is routine in our practice, and test was negative. Bacterial cultures were negative but enzyme immunoassays for toxins A and glutamate dehydrogenase (GDH) for the detection of CD infection (CDI) came back positive. The patient was started, immediately after diagnosis, on day 1 of hospitalization, on oral 125 mg of vancomycin dosed every 6 h and intravenous crystalloid rehydration therapy with 1000 mL Sodium Chloride 0.9% solution, supplemented with intravenous analgesics—Metamizole 1000 mg/2 mL twice daily.

Response to treatment was evaluated based on the dynamics of ileal output volume and clinical parameters such as urinary output volume and pain. Ileostomy volume was measured using a graded plastic recipient every 12 h, and daily total volumes were noted.

**Figure 1:**
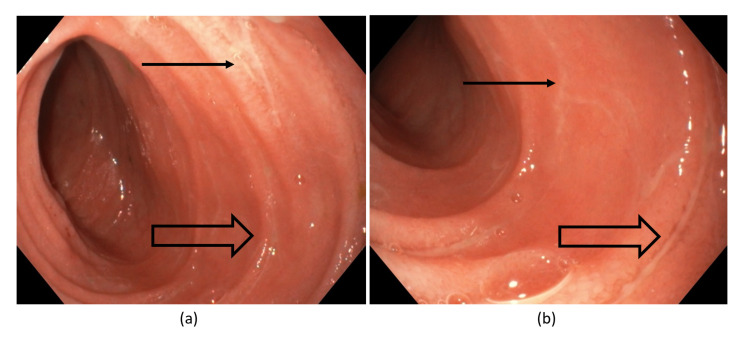
Small bowel endoscopy (**a**,**b**) showing diffuse ileal erythema with transverse superficial linear ulcerations (thick arrows) and fibrin deposits (small arrows).

Ileal endoscopic evaluation was performed by introducing the gastroscope through the ileostomy orifice and advanced approximately 30 cm upwards, revealing diffuse erythema with several superficial, linear ulcerations and fibrin deposits (Figure 1a,b).

Given the previous diagnosis of Crohn’s disease, multiple biopsies were obtained for further evaluation and differential diagnosis, to exclude an underlying active Crohn’s disease as a cause for high ostomy volumes.

**Figure 2:**
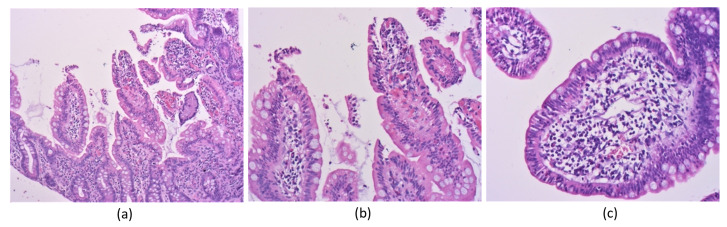
H&E stain, 10×. (**a**) Intestinal mucosa showing erosions, focal edema and moderate acute inflammatory infiltrate in lamina propria; (**b**), H&E stain, 20×. Intestinal mucosa showing superficial erosions, and focal edema and moderate acute inflammatory infiltrate within lamina propria; (**c**) H&E stain, 20×. Intestinal mucosa showing intraepithelial polymorphonuclear infiltrate, mucin depletion of the intestinal epithelium, edema and moderate acute inflammatory infiltrate within.

The histological examination concluded over an acute, non-specific, moderate severity erosive enteritis based on the absence of architectural disruptions, frequent mucosal erosions, mucus depletion, fibrin deposits and intraepithelial neutrofilic infiltrate (Figure 2).

Consequently, we continued to investigate the patient with computed tomography (CT) in order to exclude intraabdominal abscess or upstream bowel lesions of active Crohn’s disease, as causes for high output stoma, which showed a symmetric, diffuse thickening of the small-bowel wall, without obvious stenosis, without dilated enteric segments and no intraabdominal collections. The small-bowel vascularization on CT scan was negative for arterial or venous thromboses and the presence of the Comb sign was supportive of a local inflammatory process.

**Figure 3:**
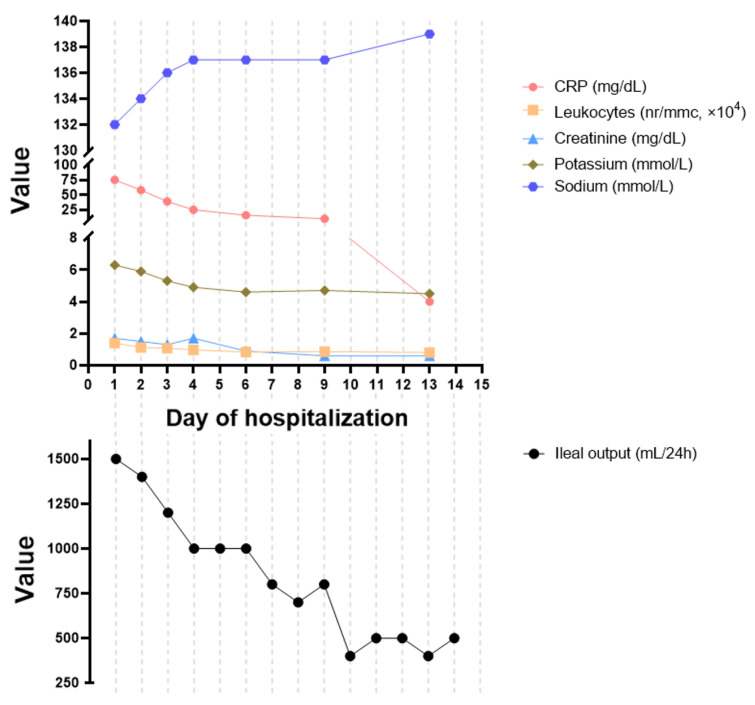
Evolution of relevant laboratory parameters and ileal output during hospitalization.

By the fourth day of treatment, the patient was rapidly recovering—the ileostomy volumes were decreasing and abdominal pain was absent. Rehydration therapy and analgesics were stopped on day 6 of treatment. In hospital evolution of altered laboratory parameters and ileal output volume are presented in Figure 3. The patient was happy to be discharged after 14 days of treatment with low-volume output (<500 mL/24 h) and normalized serum ion concentrations and renal function tests.

## 3. Discussion

We presented a case of CDE in a patient with previously diagnosed stricturing Crohn’s disease. The particular feature of our case resides on the coexistence of IBD with CDE, especially in the postoperative setting, when high-output volume of stomas is difficult to interpret, thus making differential diagnosis of utmost importance. Moreover, there is a conventional paradigm correlating CD with colitis, this contributing to delays in diagnosis and adapted management, that can negatively impact the outcome.

In our case report, the difficulty of differential diagnosis relies on the lack of previous data regarding the small-bowel extension of Crohn’s disease, upstream active disease being able to reproduce the same clinical scenario as CDE.

To further explore patient characteristics, management considerations, and outcome trajectories in patients with CDE, we conducted a literature review using the PubMed database. Key terms included “Clostridium difficile”, “Clostridioides difficile”, “small bowel”, “enteritis”, “enteral”, and “pouchitis” were identified either as medical subject heading (MeSH) terms or within the title and/or abstract. All cases published in the last 20 years were included in our review for pooled analysis. Veterinary studies were excluded, as were basic science studies and articles focusing on pediatric patients (age <18 years). Per our selection strategy, 77 reported cases were identified in 49 publications and our results are presented in Table 1 [3,4,5,6,7,8,9,10,11,12,13,14,15,16,17,18,19,20,21,22,23,24,25,26,27,28,29,30,31,32,33,34,35,36,37,38,39,40,41,42,43,44,45,46,47,48,49,50].

Within the identified cases, the following parameters were examined: age, sex, inflammatory bowel disease (IBD) status, gastrointestinal (GI) cancer history, recent hospitalization, previous surgery, predisposing antibiotic use, immunosuppression, treatment administered (conservative and/or surgical), intensive care unit (ICU) transfer, time to outcome (defined as either infection resolution of patient death), and readmission. In all patients, the diagnosis of CDE was confirmed via positive CD toxin assays and supplemented with either: (i) CT scans revealing inflammatory changes (e.g., bowel wall thickening, intramural air, etc.) localized to the small bowel or (ii) direct visualization of small bowel pseudomembranes. In some cases, the diagnosis was made postmortem on autopsy results, where there was histologic evidence of CDI localized to the small bowel. In a subset of patients who underwent restorative proctocolectomy with ileal pouch-anal anastomosis for IBD, CDE was treated as a diagnosis of exclusion as most patient did not have a colon. Out of 77 cases evaluated, 54 survived and 23 patients had a lethal outcome—the mortality rate of CDE in this pooled analysis is 29.8%.

For the survivors’ cohort, the mean age of the patients was 49.0 years (standard deviation 18.6), and of the 54 patients, 26 were male and 28 were female. For the non-survivor cohort, the mean age of the patients was 70.2 years (standard deviation 10.5), and of the 23 patients, 14 were male and nine were female. There was a slight predilection within the survivors’ cohort to have a diagnosis of IBD (57.4%)—23 (42.6%) and eight (14.8%) patients had a history of ulcerative colitis and Crohn’s disease, respectively. Virtually all patients (92.2%) suffered from hospital acquired CDE, where infection arose in a backdrop of hospitalization. Statistically significant differences between the two patient groups included age, IBD diagnosis, history of prior surgery, and ICU transfer.

Given the accentuated coexistence of IBD in CDE patients, positive CDE toxin assays should aid in contrasting CDE against a flare of IBD, especially in patients with previous GI-altering surgery. Although the endoscopy results in our patients helped cement the diagnosis, indeterminate features (e.g., superficial ulcerations, fibrin deposits) could raise suspicion for prestomial Crohn’s disease, with upstream disease also potentially explaining high-ouput from the ileostomy site. As such, predisposing history of recent hospitalization and antibiotics use, coupled with positive diagnostic tests for CD, may be advantageous for prompt diagnosis.

Surgery frequently initiated CDE (79.2%), where infection arose either immediately after proctocolectomy with ileostomy or after ileostomy takedown. In a minority of cases, patients had already undergone GI surgery and CDE arose independently of that initial hospitalization. GI procedures, which were implicated, include hernia repair, GU cancer-motivated resection, ileostomy closer, laparotomy for adhesiolysis, selective vagotomy, cholecystectomy, and anastomosis. Non-GI procedures, which precipitated CDE, include hemodialysis, nephrectomy, prostatectomy, aortic embolectomy, and pelvic evisceration. Non-surgical indications for admission, which instigated CDE, included pneumonia, urinary tract infections, closed non-displaced fractures, and soft tissue infections.

Antimicrobial agent use is a canonical catalyst for CDI through dysbiosis of colonic microbiota, which enables either seeding or spore germination in newly exposed or carrier patients, respectively. A detailed analysis of the antibiotics implicated in predisposing to CDE is summarized in Table 2.

In our review, only five patients (6.5%) developed CDE spontaneously without prior documented antibiotic exposure or recent hospitalization. The three most common cephalosporins included cefuroxime (*n* = 6), cefazolin (*n* = 6), and cefoxitin (*n* = 6) –in this review, second generation drugs of this class carried the highest risk of triggering CDE. The most common fluoroquinolones included ciprofloxacin (*n* = 6) and levofloxacin (*n* = 4). The most common ampicillins implicated included amoxicillin (*n* = 4), ampicillin (*n* = 2), and penicillin (*n* = 2). Multiple meta-analyses quantified antibiotic exposure and risk of CD infection—clindamycin is firmly cemented as the most frequently implicated antibiotic, followed by fluoroquinolones, cephalosporins, and penicillins [51,52]. For CDE, this pattern is somewhat upended, with cephalosporins being most commonly inculpated while clindamycin is significantly underrepresented. Cephalosporins are commonly given as part of preoperative prophylaxis; it is likely the high surgical admission rates of patients we reviewed reflect predisposing antibiotic use

In the majority of patients in this review, CDE arose in context of surgically altered GI anatomy—48 patients underwent colectomy with ileostomy. CD may colonize the large bowel—intestinal resection, which disrupts the ileocecal valve, may therefore facilitate bacterial translocation to the small bowel, leading to CDE [8]. However, CDE can affect patients with an anatomically normal GI tract and an intact ileocecal valve, as was reported in the case series by Lavallee and colleagues. [26]. Why certain patients suffer from a particularly deleterious progression of CD with severe features, such as ischemic colitis or enteritis is unclear [53]. Lack of immortalized appropriate cell lines (human small bowel intestinal epithelium) complicates elucidation of pathophysiologic mechanisms underlying CDE. Concomitant involvement of the small and large bowel in CDE has also been reported in 13 cases. Kurtz et al. documented a patient who underwent proctocolectomy, in addition to progressive small bowel resections due to recalcitrant Crohn’s disease—despite less than four feet of small bowel remaining, the patient still developed CDE [33].

It is challenging to accurately depict the exact treatment regimen—for most cases, the cornerstone of therapy was parenteral metronidazole with enteral vancomycin. However, it was administered with considerable variation. In some cases, antibiotic therapy was sequential, beginning with metronidazole and after several days transitioning to exclusively vancomycin. If the patients could not tolerate combinatorial therapy, they were administered intravenous fluids with metronidazole until they were able to tolerate oral metronidazole with vancomycin. For patients with stomas, vancomycin could also be administered as enemas per the distal limb of the conduit. In instances where CDE resulted in diffuse mucosal bleeding, vancomycin-soaked tamponade use was also reported. Adjunctive treatments included total parenteral nutrition, loperamide, fiber, oral fluid restriction, and in severe cases, other antibiotics were added—most commonly carbapenems.

As patients improved, there was a general trend to switch them to enteral vancomycin and continue therapy for up to four weeks in an outpatient setting. In approximately one third of patients, infection trajectory necessitated therapeutic subtotal resection of the colon and terminal ileum, in addition to antibiotics. “Unknown” treatments, as denoted in Table 1, most often referred to broad-spectrum antibiotics, which were not specified by the authors. “Other” treatments included streptomycin (*n* = 1), supportive treatment (*n* = 2) or combinatorial therapy (e.g., tobramycin, teicoplanin, or gentamicin combined with metronidazole), which were chosen to either circumvent patient antibiotic allergies or cover for a co-infection, such as pneumonia or a lower urinary tract infection. In a pediatric cohort of 18 patients (average age 4.8 years), majority of cases (72.2%) did not require dedicated treatment and were managed via antibiotic discontinuation and observation—a stark contrast to adult patients in our study, where only two patients were managed with antibiotics [50].

Grouping patients by strictly by presence or absence of prior abdominal surgery was found to be misleading, as it disrupted the temporal relationship of events that led up to the CDE infection. Majority of CDE cases arose in patients who underwent prior GI surgery, usually for IBD. However, in a minority of cases, there was history of GI surgery and therefore, altered bowel anatomy—however, hospitalization that incited CDE was unrelated to the original GI procedure. For example, a patient underwent complication-free IPAA for recalcitrant UC and six months later underwent elective hernia repair, which ultimately precipitated CDE. In order to highlight this important distinction, we additionally created the “Was CDE caused by surgery for which the patient was admitted” column. Indications for ICU transfer included hemodynamic decompensation, bowel perforation, sepsis, and multiorgan dysfunction. Virtually all patients who survived CDE were discharged in good health. One patient survived CDE, but had a complicated course and could not be weaned of ventilatory support—she was discharged to a chronic care facility. Cause of death was generally attributed to either protracted hospitalization, such as respiratory failure due to ventilator-associated pneumonia, or directly to sepsis and multiorgan failure induced by CDE.

Mortality rates for CDE demonstrate considerable variability. For case report-based pooled reviews, mortality attributed to CDE has been stabilizing at approximately 30% (Table 3).

In our review, mortality rates can be further decreased to 23.1%, if cases older than 20 years old are excluded. Ulrich et al. identified 44 cases in 855 postcolectomy patients—regarding outcome measures, only one patient expired due to CDE, leading to a mortality rate of 2% [48]. Furthermore, Park et al. retrospectively identified 18 pediatric cases of CDE—in their cohort, there were no reported deaths [50]. It can be conjectured that the mortality rate of CDE is likely lower than reported, in part due to case report bias and underreported incidence of CDE.

## 4. Conclusions

CDE becomes more frequently diagnosed possibly due to an increase in colectomy rates for different indications. There is a need for an elevated degree of suspicion to differentiate from other cause of intraabdominal sepsis like acute mesenteric ischemia, intestinal obstruction, or postsurgical complications. Its high fatality rate, even though lower than previously described, makes rapid diagnosis of utmost importance to initiate adequate treatment for better outcome.

## Figures and Tables

**Table 1:** Pooled analysis of reviewed cases, detailing differing patient characteristics in survivors and non-survivors of CDE.

	Survived CDE (n = 54)	Did Not Survive CDE (n = 23)	*p*-Value
Sex			
Male	26 (48.1%)	14 (60.9%)	0.331
Female	28 (51.9%)	9 (39.1%)	
Age (Years)			
Mean (SD)	49.0 (18.6)	70.2 (10.5)	<0.001
Median [Min, Max]	49.0 [18.0, 83.0]	69.0 [53.0, 91.0]	
Inflammatory bowel disease			
Crohn’s Disease	8 (14.8%)	1 (4.3%)	0.049
Ulcerative colitis	23 (42.6%)	5 (21.7%)	
None	23 (42.6%)	17 (73.9%)	
Gastrointestinal cancer (previously or concurrent)			
No	50 (92.6%)	14 (60.9%)	0.00157
Yes	4 (7.4%)	9 (39.1%)	
Recent hospitalization			
Surgical admission	42 (77.8%)	19 (82.6%)	0.903
Non-surgical admission	7 (13.0%)	3 (13.0%)	
No recent hospitalization	5 (9.3%)	1 (4.3%)	
History of surgery			
IPAA	25 (46.3%)	2 (8.7%)	0.0171
Total colectomy	7 (13.0%)	4 (17.4%)	
Hemicolectomy	6 (11.1%)	5 (21.7%)	
Non-GI	1 (1.9%)	2 (8.7%)	
Other	11 (20.4%)	7 (30.4%)	
None	4 (7.4%)	3 (13.0%)	
Concurrent CD colitis			
Yes	9 (16.7%)	4 (17.4%)	1
No	45 (83.3%)	19 (82.6%)	
Was CDE caused by surgery for which the patient was admitted?			
Yes	29 (53.7%)	14 (60.9%)	0.835
No, other surgery	16 (29.6%)	6 (26.1%)	
No, non-surgical	9 (16.7%)	3 (13.0%)	
Predisposing antibiotic use			
Yes	39 (72.2%)	16 (69.6%)	0.913
No	3 (5.6%)	2 (8.7%)	
Unknown	12 (22.2%)	5 (21.7%)	
Immunosuppressed			
Yes	15 (27.8%)	8 (34.8%)	0.894
No	29 (53.7%)	11 (47.8%)	
Unknown	10 (18.5%)	4 (17.4%)	
Treatment administered			
Metronidazole with vancomycin	24 (44.4%)	13 (56.5%)	0.626
Metronidazole	13 (24.1%)	4 (17.4%)	
Vancomycin	11 (20.4%)	2 (8.7%)	
Other	3 (5.6%)	2 (8.7%)	
Unknown	3 (5.6%)	2 (8.7%)	
Surgical treatment of CDE			
Yes	14 (25.9%)	9 (39.1%)	0.283
No	40 (74.1%)	14 (60.9%)	
ICU transfer			
Yes	17 (31.5%)	22 (95.7%)	<0.001
No	37 (68.5%)	1 (4.3%)	
Time to outcome (Resolution of infection or death)			
<2 weeks	27 (50.0%)	9 (39.1%)	0.766
>2 weeks	24 (44.4%)	13 (56.5%)	
Unknown	3 (5.6%)	1 (4.3%)	
Readmission			
Yes	4 (7.4%)	0 (0%)	-
No	50 (92.6%)	0 (0%)	
Not applicable	0 (0%)	23 (100%)	

CD: *Clostridioides Difficile*; CDE: *Clostridioides Difficile* enteritis; SD: standard deviation; IPAA: ileal pouch–anal anastomosis; GI: gastrointestinal; ICU: intensive care unit.

**Table 2:** Classes of antibiotics predisposing to CDE.

Antibiotic	Case Load
Cephalosporins	21 (27.3%)
Fluoroquinolones	10 (13.0%)
Penicillins	9 (11.7%)
Carbapenems	2 (2.6%)
Metronidazole	2 (2.6%)
Trimethoprim / Sulfamethoxazole	2 (2.6%)
Doxycycline	1 (1.3%)
Vancomycin	1 (1.3%)
Rifampin	1 (1.3%)
Clindamycin	1 (1.3%)
Unknown	22 (28.6%)
None	5 (6.5%)

**Table 3:** Review of historically conducted literature reviews of *Clostridioides Difficile* enteritis and the evolution of the associated mortality rate, as case number increased.

Author and Year	Cases Reviewed	Case Year Range	CDE Mortality Rate
Freiler et al., 2001 [12]	10	1980–2001	60%
Lundeen et al., 2007 [18]	20	1980–2007	45%
Holmer et al., 2011 [36]	56	1980–2011	32.1%
Beal et al., 2015 [3]	63	1980–2015	30.1%
Present study	77	2001–2021	29.8%

## Data Availability

The raw data is available at the corresponding author upon reasonable request.
